# The impact of the COVID-19 pandemic on incident cases of chronic diseases in Finland

**DOI:** 10.1093/eurpub/ckac107

**Published:** 2022-08-16

**Authors:** Katja Wikström, Miika Linna, Tiina Laatikainen

**Affiliations:** Institute of Public Health and Clinical Nutrition, University of Eastern Finland, Kuopio, Finland; Department of Public Health and Welfare, Finnish Institute for Health and Welfare, Helsinki, Finland; Department of Health and Social Management, University of Eastern Finland, Kuopio, Finland; Institute of Healthcare Engineering, Management and Architecture, Aalto University, Helsinki, Finland; Institute of Public Health and Clinical Nutrition, University of Eastern Finland, Kuopio, Finland; Department of Public Health and Welfare, Finnish Institute for Health and Welfare, Helsinki, Finland; Joint Municipal Authority for North Karelia Social and Health Services, Joensuu, Finland

**Keywords:** COVID-19, health services, health care, detection, chronic diseases, incidence

## Abstract

The COVID-19 pandemic has caused changes in the availability and use of health services, and disruptions have been reported in chronic disease management. We aimed to study the impact of the pandemic on the incidence of chronic diseases in Finland using register-based data. Incident cases of chronic diseases decreased, except cases of anxiety disorders. The annual reductions ranged from 5% in cases of cancers to over 16% in cases of type 2 diabetes. These findings may be due to diagnostic delays and highlight the importance to ensuring access to health care and the continuity of care in all circumstances.

## Introduction

The COVID-19 pandemic has affected the provision of health services on a large scale and caused changes in the availability and use of services in several countries.[1] Also in Finland the range of health services has been narrowed as resources have been shifted to deal with the COVID-19 pandemic. During the first wave of COVID-19, the Finnish Government declared a state of emergency from the mid-March to mid-June 2020 and implemented several restrictive regulations and recommendations, such as public institutions were closed and inhabitants 70 years and older were asked to stay at home. During the second wave in the autumn 2020, measures were less restrictive. However, the restrictive measures to reduce the spread of COVID-19 and protect those at risk limited people´s access to healthcare, and some patients might have hesitated to seek care due to the fear of infection.[2]

The management of chronic diseases require regular monitoring and integrated care, but due to the pandemic severe disruptions have been reported in the processes of routine care. The study by Coma et al.[3] analysed the consequences of lockdown measures on the control of chronic diseases in primary care. They found a decrease in 9 out of 10 control indicators for patients in primary care, including a decrease in LDL cholesterol and blood pressure control in ischemic heart diseases and in glycated haemoglobin A control in type 2 diabetes. Also, delays in the detection of chronic diseases, such as cancers and heart diseases, have been reported due to the sub-optimal screening and testing during the pandemic.[4, 5] The aim of this study was to examine the impact of the pandemic on incident cases of chronic diseases among the Finnish population during the first year of pandemic.

## Methods

The study population comprised all individuals aged 18 years or older who used Finnish health care services in 2019-2020. The data were extracted from the Finnish Care Register, which covers the health information on the clients treated in health centres, hospitals, and other institutions providing outpatient and inpatient care as well as on home-nursing clients. The common chronic diseases were defined from the register using ICD-10 codes for diagnosis. .  Data on the incidence of type 2 diabetes (E11), asthma (J45, J46), ischaemic heart diseases (I20-I25), cerebrovascular diseases (I60-I69, G45.9), hypertension (I10), hyperlipidaemias (E78), back pain (M54), arthrosis (M15-M17), depression (F32, F33), anxiety disorders (F40, F41), gingivitis and periodontal diseases (K05), and cancers including in-situ carcinomas (C00-C97, D00-D09) were included in the analysis. The numbers of the newly diagnosed cases found in the registers in 2020 were compared with the cases found in the previous year 2019. The comparison included all public healthcare providers and those private care providers whose data was complete for both years. Cases where a patient did not have any recordings of a diagnosis of interest from the previous year's 2015-2018 were regarded as incident cases.

## Results

In 2019, there were a total of 676 846 incident cases, from which 35 748 were type 2 diabetes cases, 29 620 asthma cases, 32 426 ischaemic heart diseases, 28 775 cerebrovascular diseases, 101 996 hypertension cases, 56 928 hyperlipidaemias, 90 044 back pain cases, 51 065 arthrosis cases, 42 248 depression cases, 38 039 anxiety disorders, 128 800 gingivitis and periodontal diseases, and 41 157 cancer cases. In 2020, the total number of incident cases (n=602 144) was 11% lower than in 2019. There were reductions in the numbers of new cases in all disease groups, except for the group of anxiety disorders where a slight increase was observed. The annual reductions ranged from 5% to 16%. Figure 1 shows the changes in the incident cases as percentages between the years.

[Insert Fig. 1]

**Discussion**

At the time of writing of this short report, Finland has been in the middle of a COVID-19 pandemic for two years. The effect of the pandemic on incident cases of chronic diseases was assessed by examining the number of new cases reported to the Finnish Care Register during 2019 and 2020. In 2020, there were a total of 11% fewer cases reported to the register than in the previous year. The study by Siso-Almirall et al. analysed the impact of prioritizing care for COVID-19 patients on the detection and care of chronic diseases and their risk factors managed in 3 primary care centres in Spain.[6] Our findings are in line with this Spanish study, which observed significant reductions in the incidence rates of cardiovascular risk factors and diseases (e.g. hypercholesterolemia and type 2 diabetes), chronic non-cardiovascular diseases (e.g. dementia and COPD), and some cancers/tumours (e.g. melanoma and colon polyps) in 2020 compared with 2017-2019. A decrease in the incidence of acute coronary syndromes during the COVID-19 lockdown has been reported also in the study by Uimonen et al. covering the catchment area of three Finnish hospitals.[7]

The decreasing trend in the numbers of new diabetes and cancer cases due to diagnostic delays during the pandemic have been reported from the UK.[5, 8] It has been estimated that approximately 60 000 type 2 diabetes diagnoses were missed or delayed in the UK between March and December 2020.[8] Globally, the mental health burden has increased during the pandemic.[9] The systematic review by Santomauro et al. indicated an increase of over 25% in cases of major depressive and anxiety disorders due to the COVID-19 pandemic.[9] Consistent with the results of Siso-Almirall et al.[6], we found a reduction in new cases of depression and an increase in the number of anxiety disorders in 2020 compared with 2019.

The main strength of our study is a rich data set including all primary and secondary health care visits of the Finnish adult population . Although some misclassification is always present in the register data, it is a feasible and cost-effective way to study a large, nationwide population. In addition, the Finnish Care Register use the ICD codes for diagnosis, and codes are recorded to the register by health care professionals. The coverage, accuracy and reliability of the Care Register for Health Care has documented previously[10]. In this study, the availability of data from prior years was crucial in defining the actual new diagnoses for 2019 and 2020. However, the data was available only until the end of the year 2020. As more data becomes available, future work should build on our analyses to monitor the long-term effects of the pandemic on the detection and management of chronic diseases, and to study the explanatory factors for the observed differences.

This study shows a temporal coincidence between the first year of the pandemic and the reduction of incident cases which may be partly explained by diagnostic delays due to changes in health services and measures limiting the access to healthcare. It is important to evaluate the impact of the pandemic on diagnostic delays after a longer follow-up period and evaluate their possible, long-term health consequences. From a public health point of view, the early detection of chronic diseases is important both during and after the COVID-19 pandemic. Access to health services or alternative services must be secured also in exceptional circumstances and restrictive measures must not be an obstacle for the diagnosis of diseases and the implementation of good care.

**Funding:** This study was partly funded by the Strategic Research Council of the Academy of Finland [project IMPRO 335524, 336325 & 336329].

**Conflicts of interest:** None declared.

**Key points:**

- The number of new cases of chronic diseases decreased in Finland during the first year of the pandemic.
- The annual reductions ranged from 5% in cases of cancers to over 16% in cases of type 2 diabetes.
- As an exception to this general trend, a slight increase was observed in the incidence of anxiety disorders.
- The decreasing incidence may reflect delays in diagnostic care due to changes in the availability and use of health services during the pandemic.
- In all circumstances, access to health care and the continuity of care need to be secured.

Figure 1. Changes in incident cases of chronic diseases during the first year of the COVID-19 pandemic

(Figure in tiff. format is included as an attachment)
